# A Nationwide Survey of UK Oncologists' Views on the Choice of Radiotherapy Regime for the Reconstructed Chest Wall in Breast Cancer Patients

**DOI:** 10.1155/2017/6385432

**Published:** 2017-01-01

**Authors:** Nicola Davis, Rema Jyothirmayi

**Affiliations:** Kent Oncology Centre, Maidstone Hospital, Maidstone, UK

## Abstract

*Aims*. This paper describes a UK survey of the choice of radiotherapy regime for the reconstructed chest wall in breast cancer patients. Questions focused on which fractionation regime consultants choose, their reasons for this, whether the type of reconstruction influences their choice, and whether bolus is used in patients who have undergone immediate reconstructive surgery.* Materials and Methods*. Between July 2014 and July 2015 a survey was sent by email to UK consultant radiation oncologists treating breast cancer.* Results*. The response rate was 73%. 67% of respondents use 40 Gray (Gy) in 15 fractions, with 22% using 50 Gy in 25 fractions and 7% using other regimes. For 90% of consultants the type of reconstruction did not influence their decision regarding choice of fractionation. 83% of respondents do not usually use a bolus for chest wall radiotherapy in patients who have had immediate reconstructive surgery.* Conclusions*. This survey illustrates there is variation in practice in the management of patients with breast cancer who have undergone immediate reconstructive surgery in the UK. There is a need for further research to determine which fractionation regime is optimal, whether the type of surgery is relevant, and whether bolus should be added.

## 1. Introduction

Postmastectomy radiotherapy has been shown to reduce locoregional recurrence and improve overall survival in patients who have high risk breast cancer [1, 2]. These patients are classified as high risk if they have T3/T4 tumours and/or ≥4 positive lymph nodes [1, 3]. Radiotherapy usually commences approximately four to eight weeks after the mastectomy [4].

Women who have had a mastectomy may be offered reconstructive surgery, the benefits of which include improvements in body image and psychological wellbeing [4, 5]. For some patients reconstruction is undertaken in the same operation as the mastectomy (immediate reconstruction), but for others this is performed as a separate procedure (delayed reconstruction) [2]. Data from the 2011 National Mastectomy and Breast Reconstruction Audit shows that, in the UK in 2008-9, 16,485 women underwent mastectomy, and of these 21% had a concurrent immediate breast reconstruction [6].

Based on data from the START trials, 40 Gy in 15 fractions has been accepted as the standard regime for postmastectomy radiotherapy in the UK [7]. The trials' eligibility criteria, however, excluded patients undergoing immediate surgical reconstruction, making it difficult to apply the data to this subgroup of patients. Moreover, although common indications for using bolus include close or positive margins, skin involvement by the tumour, and tumour size [5], the role of using a bolus in treating the reconstructed chest wall is also unclear [8]. There have been no randomised trials evaluating the effects of radiotherapy or the influence of surgical techniques on complications or on the cosmetic outcomes of breast reconstruction [2, 9].

There is no national guidance on the radiotherapy fractionation for treatment following reconstructive surgery. This survey was therefore undertaken to evaluate the variation in clinical practice across the UK. The goals of this study were

- to identify the fractionation regimes that are chosen for the reconstructed chest wall and the reasons why these are selected;
- to consider whether the type of reconstruction influences the choice of regime and if so why;
- to evaluate whether bolus is used when treating the chest wall in patients with breast cancer who have had immediate reconstructive surgery and the reasons for these choices.

## 2. Methods

An original electronic survey questionnaire was constructed and distributed via email, with an introductory section explaining the purpose of the study, to UK radiation oncology consultants treating breast cancer. Attempts were made to identify consultants' email addresses from the employing hospital's websites and other Internet searches. Despite this there were notable difficulties obtaining a complete database of email addresses. Therefore, the survey was also sent out using a web link to the email distribution list of the UK Breast Cancer Meeting (a national UK meeting of oncologists treating breast cancer). The survey was sent out multiple times between July 2014 and July 2015, to improve the response rate.

The questionnaire consisted of six questions, most of which required respondents to choose from preselected options, with descriptive answers required for the questions requesting an explanation for the choices made (see the Appendix).

## 3. Results

### 3.1. Response to Questionnaires

Using the Royal College of Radiologists' most recent census fifty-nine oncology centres were identified, with the addition of Worcester which had been established since then. There were a total of seventy-three responses from forty-four centres (73% response rate), and of these fifteen centres had responses from more than one consultant (see Table 1).

### 3.2. Choice of Radiotherapy Regime

Forty-nine respondents (67%) use 40 Gy in 15 fractions, 16 respondents (22%) use 50 Gy in 25 fractions, and three respondents commented that both regimes were used. The remaining five respondents (7%) chose other regimes: 50 Gy in 28 fractions, 45 Gy in 25 fractions, and 45 Gy in 20 fractions (See Table 1).

In terms of the reasons for choice of regime, the descriptive answers have been analysed and common themes are as follows, with representative quotes.

For 40 Gy in 15 fractions the most common explanations were that this is considered to be the standard regime and has been historically practised and therefore been incorporated into many departments' protocols. Another recurring explanation was that the choice is evidence based, with the START trial frequently mentioned, as well as consideration given to the underlying radiobiology in many respondents' answers. For example, one consultant commented that their practice had* “switched away from 50/25# with START data (although we recognise that immediate reconstructed group were excluded from the trial). Data from the START trial suggested that 40/15# was better for breast late effects and so even if worse in the reconstructed breast may well be effectively isoequivalent.”* Another reason given for the choice of 40 Gy in 15 fractions was radiotherapy capacity with one respondent commenting on* “pressure on fraction numbers.”* There were also quite a few comments such as* “no evidence to suggest different regime anything better.”*

The lack of impact on cosmesis was also highlighted in many answers. For example, one consultant commented that* “fractionating radiotherapy further does not seem to change cosmetic outcome” *while another stated that* “after discussion with our breast surgical colleagues we have decided that the cosmetic outcome should not be compromised with this regime compared to 50 Gy/25#.*” For 50 Gy in 25 fractions the most common explanation for choosing this regime related to concerns about cosmesis, for example,* “to reduce the chance and extent of scarring,” *being* “assumed to be less likely to cause delayed fibrosis,”* and* “presumption of better cosmetic result.”* Many respondents also commented that this regime was selected for historical reasons and was current standard protocol in their department. Moreover, there was also mention by one consultant that this regime was chosen as a* “specific request from surgeons for anyone with tissue transfer or implant.”*

For the three respondents that use both 40 Gy in 15 fractions and 50 Gy in 25 fractions the reasons given were that it depended on the type of surgery with one consultant commenting that* “if simple op then 40 Gy, if complex then 50 Gy, unhappy to use 40 Gy that is, large fraction per day with fancy vascular and imported skin/muscle type procedures”*.

With respect to the other regimes, the respondent who chose 50 Gy in 28 fractions uses this because they are* “worried about late effects with increased risk of fibrosis,”* for 45 Gy in 25 fractions, this is selected in order* “to reduce late effect”* and because of* “lower dose per fraction,”* and, for 45 Gy in 20 fractions, the explanations for this choice were given as* “departmental discussion and consensus”* and “*lower dose per fraction to spare reconstruction*.”

### 3.3. Type of Reconstruction

For sixty-six consultants (90%) the type of reconstruction did not influence their decision regarding choice of fractionation (see Table 2). The most common reason given for this decision was that there was no evidence; one consultant commented that there was* “no evidence that autologous tissue transplanted into the area would have different radiobiology”* while another noted that there is an* “assumption that all reconstructive surgery may be affected by post radiation sequelae.*” Other comments included that* “dose homogeneity more important”* and that* “patient's biology has more effect on response than type of reconstruction.*” For the six respondents (8%) who answered that the type of reconstruction was influential on their choice of fractionation (see Table 2), the reasons related to concerns about cosmetic outcome and achieving dose constraints.

### 3.4. Use of Bolus

Sixty (82%) respondents answered that they did not usually use a bolus when treating the chest wall in patients with breast cancer who have had immediate reconstructive surgery (see Table 3). Many respondents felt that there was no evidence for the use of a bolus after reconstruction, and many suggested that the reconstructed skin is itself a bolus. Several comments also related to concerns about a bolus worsening side effects particularly in terms of cosmesis.

Although a bolus is not used routinely, respondents' answers identified common indications for its use as follows: positive margins, skin involvement, lymphovascular invasion, and an inflammatory cancer. In addition, there was an appreciation that the use of a bolus may be necessary depending on the dose distribution with one consultant noting that* “recently I have used bolus with sub pectoral implants as the 95% was sitting within the implant and not covering the anterior tissue.”*

For the eleven respondents (15%) that routinely use a bolus (see Table 3) the most common explanations for this related to concerns about dose distribution and the skin being at risk of recurrence. One consultant commented* “I always use a bolus – to treat without gives 80% of expected dose to area requiring treatment (3*–*10 mm).”*

### 3.5. Other Comments

The last question asked respondents for any other comments regarding chest wall radiotherapy in breast cancer patients who have had immediate reconstructive surgery. These have been analysed and some common themes are as follows.

For many respondents a need for further research has been recognised:

- “Would be very interested if anyone else has found data and in the results of the survey.”
- “Will be very interested to see others' practice. Relative paucity of helpful data to guide sensible decision making.”
- “Would like to do a trial of 40 Gy in 15 fractions versus 50 in 25.”
- “There is a need for consensus regarding indications for bolus post reconstruction.”
- “I think there is still too much myth and superstition amongst the surgical population about radiotherapy. I think the improved surgical and radiotherapy techniques mean that the complications previously seen are much less frequent. A prospective database would be welcome using the same annual CRF that we use in START and FAST.”

Some respondents commented that in their centres few patients who have immediate reconstructive surgery would need radiotherapy, due to patient selection. There also seemed to be concerns about the complexity of reconstructive surgery and how this influences decisions about radiotherapy:

- “Concerns regarding dose distribution especially around port. Concerns about target volume coverage.”
- “Sometimes the cosmetic result is not as good with radiotherapy after implant based reconstruction rather than tissue transfer.”
- “Only concern is use of tissue expanders with magnetic inflation ports. Uncertainty about dosimetry so have asked our surgeons to avoid. If patient wants immediate recon I simply warn them of possible contracture and consent.”
- “RT plans sometimes need to be compromised too much heart/lung to cover CW/reconstruction, artefact from the valve in immediate delay reconstruction. Negative cosmetic impact if radiotherapy. Intermediate risk patients bias.”

Because of these concerns one consultant commented that there needs to be* “good discussion with oncoplastics prior to RT”*.

Four respondents commented that they would prefer to delay reconstruction if possible:

- “Ideally should pre-empt need for RT and offer before recon if possible and wait at least a year before recon.”
- “I would rather avoid by pre selecting patients at high risk for delayed reconstruction.”

## 4. Discussion

Chest wall reconstructive surgery can involve a number of different techniques including autologous reconstruction utilising the patient's own tissues, synthetic implants, or temporary tissue expanders which can be placed initially and later replaced [2]. There are also various new devices being used such as internal magnetic metallic ports which may be placed within temporary tissue expanders [4, 5]. The risks of undergoing implant based reconstruction include scarring at the interface between the implant and the tissue, capsular contracture, infection, pain, skin necrosis, fibrosis, and impaired wound healing, and the chance of these complications developing seems to be higher in patients who also receive radiotherapy [2, 10]. The risks of autologous tissue reconstruction include fat necrosis, fibrosis, atrophy, and flap contraction, but some studies indicate that this technique produces better results with radiation compared to what occurs with implant based techniques [2, 11, 12]. For both types of surgery these effects may cause significant morbidity and potentially require repeated procedures, with associated psychological distress [2]. Chen et al.'s 2010 study exploring the perspectives and practice of radiation oncologists treating patients with chest wall radiotherapy found that 57% of participants felt* “that reconstruction challenges their ability to deliver effective breast post mastectomy radiotherapy”* [5].

There are also concerns about how reconstruction may affect dose heterogeneity within the radiation field. For example, in Chen's study 66% of physicians agreed that the volume of fluid within an implant affects radiation dose distribution and can make treatment planning challenging [5]. Furthermore, 39% of respondents to Chen's survey requested a moderately sized expander in order to minimise dose to critical structures including the heart and lungs [5]. There is therefore a challenge in balancing the benefits and risks of both reconstruction and radiotherapy as part of the management of patients with breast cancer [2].

With respect to this survey, it was not possible to obtain responses from all UK oncology centres, so that the results may not therefore be entirely representative of current practice. This is a reflection in part of the difficulty the authors had in obtaining the names and email addresses of radiation oncologists treating patients with breast cancer. In addition, as was noted by certain respondents practice is constantly changing, and therefore some clinicians may already have altered their management since completing this survey.

The results of this survey highlight a significant variation in practice across UK oncology centres in terms of the use of radiotherapy following reconstruction surgery for breast cancer patients. While the majority of consultants use 40 Gy in 15 fractions, many consultants prefer to use 50 Gy in 25 fractions. The results also indicated that other regimes are also used, highlighting the lack of consensus on which fractionation should be delivered within this context. Furthermore, when analysing the results there was also variation in practice within oncology centres, and this was even mentioned by several respondents; for example: “*not all consultants treating breast cancer in my centre share my views*” and* “2 clinicians disagree.”*

The survey also highlighted that the complexity of surgery influences clinicians' treatment decisions, and hence multidisciplinary team involvement is key.

## 5. Conclusions

The results of this survey highlight the need for further research into the use of radiotherapy to the reconstructed chest wall for breast cancer patients. Although many respondents used the START trial [7] as their evidence base for their choices, patients who had undergone reconstructive surgery were not included in this trial.

A national UK study should be considered using retrospective data to compare fractionation regimes, to gather evidence prior to undertaking a formal randomised controlled trial.

## Figures and Tables

**Table 1:** Choice of fractionation.

Fractionation	Number of responses
40 Gy in 15#	49 (represented by 32 different centres)
50 Gy in 25#	16 (represented by 8 different centres)
Both 40 Gy in 15# & 50 Gy in 25#	3 (represented by 3 centres)
Others—45 Gy in 20#	3 (represented by 2 centres)
Others—50 Gy in 28#	1
Others—45 Gy in 25#	1

**Table 2:** Responses to the question “does the type of reconstruction influence your decision regarding choice of fractionation?”

Response	Number of responses
No	66
Yes	6
No answer	1

**Table 3:** Responses to the question “do you usually use a bolus when treating the chest wall in patients with breast cancer who have had immediate reconstructive surgery?”

Response	Number of responses
No	60
Yes	11
Yes and No	2

## Appendix

### Questionnaire

(1) Which oncology centre do you work in?
(2) Which radiotherapy regime do you usually use for treating the chest wall in patients with breast cancer who have had immediate reconstructive surgery?
  (i) 40 Gy in 15 fractions
  (ii) 50 Gy in 25 fractions
  (iii) Other (please specify)
(3) Please explain why you choose this particular regime?
(4) Does the type of reconstruction influence your decision regarding choice of fractionation?
If no, why?
If yes, why?
(5) Do you usually use a bolus when treating the chest wall in patients with breast cancer who have had immediate reconstructive surgery?
Yes
No
(6) Do you have any other comments regarding chest wall radiotherapy in breast cancer patients who have had immediate reconstructive surgery?

## References

[1] Mukesh M. B., Duke S., Parashar D., Wishart G., Coles C. E., Wilson C. (2014). The Cambridge post-mastectomy radiotherapy (C-PMRT) index: a practical tool for patient selection. *Radiotherapy and Oncology*.

[2] Jagsi R. (2013). Postmastectomy radiation therapy: an overview for the practicing surgeon. *ISRN Surgery*.

[3] Ribuffo D., Monfrecola A., Guerra M. (2015). Does postoperative radiation therapy represent a contraindication to expander-implant based immediate breast reconstruction? An update 2012–2014. *European Review for Medical and Pharmacological Sciences*.

[4] Trombetta D. M., Cardoso S. C., Alves V. G. L., Facure A., Batista D. V. S., Da Silva A. X. (2015). Evaluation of the radiotherapy treatment planning in the presence of a magnetic valve tissue expander. *PLoS ONE*.

[5] Chen S. A., Hiley C., Nickleach D. (2013). Breast reconstruction and post-mastectomy radiation practice. *Radiation Oncology*.

[6] The NHS Information Centre (2011). *National Mastectomy and Breast Reconstruction Audit*.

[7] Haviland J. S., Owen J. R., Dewar J. A. (2013). The UK Standardisation of Breast Radiotherapy (START) trials of radiotherapy hypofractionation for treatment of early breast cancer: 10-year follow-up results of two randomiLancet Oncologysed controlled trials. *The Lancet Oncology*.

[8] Vu T. T. T., Pignol J.-P., Rakovitch E., Spayne J., Paszat L. (2007). Variability in radiation oncologists' opinion on the indication of a bolus in post-mastectomy radiotherapy: an international survey. *Clinical Oncology*.

[9] Lee M., Reinertsen E., McClure E. (2015). Surgeon motivations behind the timing of breast reconstruction in patients requiring postmastectomy radiation therapy. *Journal of Plastic, Reconstructive and Aesthetic Surgery*.

[10] Whitfield G. A., Horan G., Irwin M. S., Malata C. M., Wishart G. C., Wilson C. B. (2009). Incidence of severe capsular contracture following implant-based immediate breast reconstruction with or without postoperative chest wall radiotherapy using 40 Gray in 15 fractions. *Radiotherapy and Oncology*.

[11] Barry M., Kell M. R. (2011). Radiotherapy and breast reconstruction: a meta-analysis. *Breast Cancer Research and Treatment*.

[12] Jhaveri J. D., Rush S. C., Kostroff K. (2008). Clinical outcomes of postmastectomy radiation therapy after immediate breast reconstruction. *International Journal of Radiation Oncology, Biology, Physics*.

